# On Certain Practical Diagnostic Distinctions between the Concentrated Endemic, Commonly Called the Yellow or Bulam Fever of the West Indies, and the Marsh Remittent Fever

**Published:** 1819-01-01

**Authors:** John Niell

**Affiliations:** Surgeon in the Royal Navy


*On certain Practical Diagnostic Distinctions between the concentrated Endemic, commonly called the Yellow or Bulam Fever of the West Indies, and the Marsh Remittent Fever.* By JOHN NIELL, Esq. Surgeon in the Royal Navy

| Yellow or Bulam Fever. | Bilious Remittent Fever. |
|---|---|
| 1. THE invasion so sudden, that it appears as if the patient had been knocked down by a blow. | 1. The invasion slow; the symptoms aggravated by degrees. |
| 2. If the patient appear early, before reaction, the pulse is scarcely above natural in frequency, and sometimes much under it, as low as 48 pulsations in the minute. | 2. If the patient is seen early, before reaction, the pulse is so frequent that it cannot be enumerated, upwards of 130 in the minute; and the heart can be seen to beat with great violence against the ribs. |
| 3. The animal functions are greatly oppressed, the features much shrunk, betraying great anguish and distress, similar to what we often perceive in the last stage of cholera morbus. | 3. The animal functions, and features, are much as in the other species. |

Yellow or Bulam Fever.

4. The head-ache at first seems the most violent and distressing symptom, which gives an essential character to the disease; it is confined to a mere line, drawn from temple to temple, intersecting the frontal sinuses, with an acute pain in the orbits, greatly aggravated on motion, the admission of light, or looking attentively at objects, from the first moment of attack, and previous to reaction or a determination to the brain.

5. The tongue, I think I may affirm pretty positively, furnishes us with a diagnosis: in this it is clear, smooth, the papillæ never erected; of a shining, bright red appearance; moist; it may be sometimes covered with a very thin bluish white pellicle, so extremely attenuated as to permit the bright red shining aspect to appear thro' it. With this moist state of the tongue, there is great thirst; so irresistible is the propensity to indulge, that it cannot be, by any resolution in the strongest minds, effectually opposed.

6. The stomach, at first, does not generally create much uneasiness; but, on gently pressing the epigastrium, a considerable degree of pain is excited; at this period, there is seldom any gastric irritability, which, however, appears as the disease advances.

Bilious Remittent Fever.

4. The head is but little affected with pain, previous to reaction; but seems more confused, the pain confined to the forehead, and extending backwards to the coronal suture, when it becomes the object of remark. The pain in the orbits scarcely perceptible until an obvious determination to the head is manifest.

5. The tongue has always, more or less, the aspect of a morbid secretion; it is always foul; either white, dry, or furred with a yellow brown colour, from the beginning: as the disease advances, it becomes more extensively foul, and the teeth are covered with black tenacious sordes, adhering with great firmness. Though the tongue is often dry and parched, yet the thirst is very trifling; drink seldom craved, and taken with indifference when presented.

6. The stomach is generally irritable from the beginning, with copious ejections of bilious matter; even on pressing the epigastrium, little or no pain is excited in this organ.

| Yellow or Bulam Fever. | Bilious Remittent Fever. |
| --- | --- |
| 7. No remissions to be perceived; but appears to terminate its course in one protracted paroxysm. | 7. Remissions perceptible, even in the worst cases, in the first days; they, however, become more obscure as the disease advances. |

In the *second* stage, that is, when re-action appears, the symptoms of both are very similar; and the only diagnostic phenomenon now, independent of remission, is the tongue as already described. The pulse, in one case, augments its frequency, while in the other its velocity is diminished, until both come nearly to the same point of action, which is generally from about 106 to 110 in a minute. The pulse is, in both cases, now full, strong and hard; the face begins to fill, becomes inflamed; soon after this, strong and dangerous determinations of blood take place to the head and other important organs. The skin is also hot, dry, and pungent, imparting a burning disagreeable sensation to the head in health; the head-ache, in both, becomes more aggravated, until coma and delirium lull him into an insensibility of his sufferings. At the earliest period of this state, the application of means, to subdue these morbid motions, ought to be carried into effect.

In some anomalous cases, the tongue may be foul, from more organs being affected than what I consider necessary to constitute a pure case of the endemic fever of this country; but even here, there is a propensity in the tongue and teeth, if previously covered with sordes, to become clean spontaneously, before the extinction of life.

The clean state of the tongue I consider a distinctive symptom, which first arrested my attention in this fever, as it was new to me, and not usually witnessed in the fever belonging to other tropical latitudes; and I have uniformly observed this state of the tongue to point out a disease that would terminate fatally, with or without black vomit, if not subdued by the most prompt and powerful means.

Dissections in these fevers exhibit always a difference, which corresponds with external phenomena, and which I consider diagnostic. In the *remittent,* therefore, the biliary organs appear the principal foci of disease; while in the *Bulam,* the morbid derangement is confined to the villous coat of the alimentary canal, particularly the stomach.

In the *remittent fever* too, the liver is of a dark chocolate colour; it is much enlarged, and turgid, with a fluid apparently fit for being converted into bile; the gall-bladder is generally distended with an inspissated fluid, having much the appearance and consistence of tar or molasses.

The reverse of this is to be observed, on dissection, from *yellow fever;* the liver is of a light pale colour, similar in appearance to an old dried sponge, diminished in size; the gall-bladder, always containing but little bile, generally shrunk; and so much so in some cases, that it was at first supposed absent. From being shrivelled into that depression, in the superior part of the liver, and from the pale, dry appearance of the liver, we might have conjectured the principal seat of morbid destruction to be in the internal parts of the stomach and intestines.

These appearances I have observed uniformly to attend these different states as described; and I think the tongue serves as an index, at an early period, of the particular organ affected: and in anomalous cases, where the tongue is foul and loaded, having a propensity to cleanse spontaneously, it is indicative of the inflammation being more extensive. The liver may be affected, and enlarged, by morbid determinations to the biliary system, as well as a derangement of the internal tunic of the stomach. In this variety, however, I am not so fully satisfied that both organs are uniformly affected, as in that state of the disease accompanied with the red, smooth, moist, clean tongue, where the liver is found of a light brown colour, devoid of bilious matter, which, I have observed, is always an index of an inflamed and disorganized state of the villous tunic of the stomach, as well as an invariable concomitant of a most aggravated form of disease.

According to my observations, the tongue furnishes us with a diagnosis, and the last described state points out an inflammatory action, perhaps of a peculiar kind, in an extensive membrane of extreme delicacy, and immediate importance to life; and in its destruction or disorganization is involved the death of the patient. The experienced physician will find this peculiarity of the tongue connected with a primary affection of the internal tunic of the alimentary canal.

These observations are deduced from a number of facts, demonstrated by dissection, as being concomitant, which induce me to draw the following conclusions: viz. that the clean tongue is a certain diagnostic symptom, at an early period, of a most malignant disease, having its seat in a delicate membrane of the first importance to our existence, and which generally terminates, when fatally, with black vomit; that in all other cases, where the tongue is foul, and loaded with black or brown sordes, I have also observed the biliary organs to be the seat of disease; the liver displaying the effects of strong determinations; and where there is no propensity in the tongue to cleanse spontaneously, I have not had occasion to witness the villous coat of the alimentary canal exhibit marks of morbid action. But in cases, which I have denominated anomalous, indicated by the foul tongue, with a disposition to cleanse spontaneously, as the disease advances, I have likewise found the villous coat of the stomach and intestines strongly under the influence of derangement. Though the liver did not seem to partake so much of disease as in the second species, yet it always bore, more or less, evident marks of morbid determinations when contrasting it with that species of disease which has the clean tongue.

In all my experience, in the fevers of India, I never found the phenomena and dissection to correspond with those of the second order or species; and they, in every case, maintained a similar uniformity as to symptoms *before*, which accorded with appearances *after* death; but none of my observations, during my residence in that country, can induce me to recall to my remembrance, the having seen or heard of a case of that species of fever which often terminates in death, after exhibiting what is emphatically called black vomit, like coffee grounds.

Had these two fevers possessed an identity, we cannot well account, I think, for the general absence of this symptom characteristic of the yellow fever in other tropical latitudes, and its great frequency in the West Indies. It cannot be owing to the gradual change during a long voyage from a cold to a hot climate, that gives the East this exemption, because we not unfrequently meet with it in this country, in individuals who have never been out of the atmosphere of these islands; however, the great liability to be attacked by this peculiar form of fever, if an identity exist, is certainly soon after our arrival from the temperate zone. Hence, for the above reasons, this inference may be made; that the disease must depend on some peculiarity in the atmospheric temperature, or in the climate of the new world, or something connected with it, which does not generally prevail in other tropical latitudes.

### Treatment.

This consists in a modified employment of those means which experience has, for some years, stamped with success; viz. blood-letting, purgatives, and calomel. These remedies are to be used in the above order, unless where the patient is seen before re-action takes place, where the skin is cool and cadaverous-looking, harsh, and disagreeable to the touch; the countenance pourtraying great anxiety, anguish, and distress. Here it will be necessary to give a brisk purgative of calomel and jalap, that may operate before arterial excitement takes place.

In respect to blood-letting, when the head-ache is great, with pain in the eye-balls, aggravated by motion, pressure, light, or looking at any object; and where the skin is hot and dry, I direct the patient, if possible, to sit up, and in this position I abstract blood, through a moderate orifice in the arm, until the heat of the skin is uniformly reduced to the temperature of health, which is well enough ascertained, for every purpose we require, by comparing it with our own. The left axilla and side of the neck being considered as retaining most permanently the morbid temperature, we have selected these parts to direct us, and blood is drawn off until they are of the same temperature as the hand of the practitioner, in a state of perfect health. Should deliquium or syncope appear before the required effect is produced, the patient is to be laid in the horizontal position, and blood abstracted until morbid heat is removed, when a profuse, clammy, cold perspiration comes on, which may last several hours, before a gentle genial warmth returns, and convalescence may from this moment be dated; the patient being relieved from his sufferings, sinks into a sound refreshing sleep; and on awaking, finds himself perfectly easy and comfortable, with a return of the healthy action of the digestive organs. If one bleeding be large, before the temperature of the body is reduced, it is generally perfectly sufficient for the reproduction of health; but, on the other hand, if he bear it badly, that is, if these effects shall be produced by a small quantity of blood, one or more repetitions of the evacuation will be requisite before we can succeed in permanently subduing the morbid action, owing to the small quantity abstracted being inadequate to the subduction of the existing inflammation. We ought to bear in mind, that as soon as the morbid heat returns, the sooner we resort to the evacuation the better: and instead of making another aperture, the same orifice may be repeatedly opened with impunity. In order to exhibit what promptitude is requisite, and the quantity of blood that may be abstracted on these occasions, I may observe, that a thin delicate lad, about sixteen years, attacked with this ardent fever, was bled at three different periods, all in the course of ten hours, to the extent of seventy ounces, before the disease was subdued ; and in the same fever, in adults, we have repeatedly abstracted eighty ounces of blood at once, before we could produce, of course, the desired effect. On no occasion, in some hundred cases in this squadron, have we regarded the quantity of blood abstracted, further than what curiosity may have prompted ; our object was to procure a given result, and when once attained, our views were so far fulfilled ; but stopping short of this, however large the quantity may be, experience has fully demonstrated to us, that we had failed in our duty, and so far debilitated, without overcoming the force of disease ; by weakening the citadel, we were taking the most efficient means of strengthening the enemy. I therefore maintain, in the ardent fevers of this country, that stopping short of this effect, whatever the quantity may be, is not vanquishing the disease, but doing much worse—debilitating the patient.

I have observed, that although, on our first arrival in the West Indies, we bled largely, (to syncope and the relief of symptoms) yet, without attending to its effects on the surface, our efforts, on most occasions, were fruitless, our means being inadequate to the removal of a disease, whose violence and rapidity we were not sufficiently acquainted with ; requiring, therefore, a greater energy of action on our part, to afford the smallest hopes of combating it on any thing like equal terms.

In all cases where this remedy has been thus systematically used, convalescence was immediately the result, if the first bleeding had been extensive before the effect, I have endeavoured to inculcate as requisite, is produced ; and if two or more bleedings become necessary, we have convalescence generally established within the first twelve hours after the earliest appearance of reaction. This is not submitted to your perusal without extensive experience, and a firm conviction resulting therefrom of the urgent necessity of carrying this evacution to the point held out, which has been corroborated in a vast number and variety of cases, ages, and constitutions, marked by a success truly gratifying to every surgeon in this squadron, as it abridges the morbid state, human sufferings, and the labour and anxiety of the practitioner ; circumstances which of themselves are of some consideration, during the prevalence of sickness on board ship. By such prompt and decisive mode of acting, the patient is, in a few hours, rescued from a disease the most violent in its kind; convalescence is established, and recovery so rapid, that patients who have lost upwards of one hundred ounces of blood, have been at duty again before the expiration of the week. After convalescence is once established, and the functions of the stomach returned, the patient should be particularly cautioned in indulging, at least until the craving for food is strong; it should be given in small quantities, and that of the easiest digestion selected. It is out of place to observe the necessity of keeping an attentive eye to a lax state of the bowels, but for the sake of remarking that it is greatly facilitated by carrying the evacuation to the extent above described.

The alvine excretions have been almost on all occasions so copious, from the united efforts of cathartic medicine and enemata, that the patient and practitioner have been often astonished at the extent of these fœtid and fæculent discharges, which could not, I am confident, be procured without this previous relaxation by blood-letting.

It will be my object, at some future period, to endeavour to prosecute this inquiry further, and on a more extended scale, than the limits of a letter, already too long, will permit; and to show the discordancy existing among authors, relative to the propriety and extent of this evacuation. It is not to be wondered at, that such discrepancy should exist in practice, from the term fever and its dreadful consequences, debility and putrescency, being so generally applied to every morbid motion attended with much vascular action, without any evident topical affection. Hence, practitioners have been often induced to prescribe for the name, rather than attempt to trust to the evidence of their own senses, from phenomena exhibited before, and appearances which morbid anatomy displays after death—from veneration to popular opinion, which is often founded in error, by making every effort to subdue an inflammatory action which has acquired such a control over the sensorial energy as to exhibit symptoms of great and direct debility, which that accurate observer, Dr. Clarke, in his long Voyages, calls an engorgement of the brain ; and without viewing it as an effect of a strong impression on the nervous system, he made his means subservient to his hypothesis, by introducing bark, wine, &c. to overcome this cerebral oppression, or strong determination, instead of using the lancet, which some of his cases seemed urgently to demand. These remarks may appear harsh, and unbecoming a member of a liberal science; but, as the experience of others clashes so much with our own, I have been often insensibly betrayed into an energy of expression which I could not well avoid, without any disrespect to preceding writers.

There are a few, indeed very few, besides yourself, who have used this remedy as it ought to be, early and boldly, or not at all; and as I consider the skin as affording, on all occasions, indications to guide us, with an accuracy which cannot admit of any arbitrary import, and of which, from description, every man can form a correct notion, I have therefore selected this as the grand leading mark in conducting this most essential evacuation, instead of "constitution, strength of the patient, &c." and the various arbitrary denominations given to arterial pulsation, which are always vague, and difficult to be understood, without experience. Even the word debility has a very equivocal meaning, for some patients apparently under this state, gain strength as the blood escapes from the vein. Under such circumstances, were bark, wine, &c. exhibited, we should expect to meet with delirium, coma, and this engorgement of the brain; but where the lancet is largely, and even unsuccessfully employed, it does not then abridge the period of human existence, though it render the road to death tranquil, collected, and easy: this even is a desirable thing when unsuccessful.

I ought to apologize for this tedious and prolix epistle, had I room; however, accept the will for the deed, and believe me to be, with much respect, regard, and esteem, yours truly, J. NIELL.

